# CX3CL1: a potential chemokine widely involved in the process spinal metastases

**DOI:** 10.18632/oncotarget.14773

**Published:** 2017-01-20

**Authors:** WangMi Liu, Chong Bian, Yun Liang, Libo Jiang, Chen Qian, Jian Dong

**Affiliations:** ^1^ Department of Orthopedic Surgery, Zhongshan Hospital, Fudan University, Shanghai, China

**Keywords:** CX3CL1, metastasis, spine

## Abstract

**Objectives:**

The aim of our study was to investigate the general mechanism of spinal metastases from five different primary cancers: lung cancer, breast cancer, liver cancer, prostate cancer, and kidney cancer.

**Results:**

From microarray analysis and validation by real-time polymerase chain reaction, CX3C chemokine ligand 1 (CX3CL1) appeared to be a potential chemokine widely involved in the process of spinal metastases. Further studies revealed that, compared with normal controls, serum samples from patients with spinal metastases from the lung (*P* < 0.01), kidney (*P* < 0.05) and prostate (*P* < 0.05) contained significantly higher levels of CX3CL1, whereas those from patients with spinal metastases from the liver and breast had a tendency to contain higher levels of CX3CL1 but without significance. Immunohistochemical staining for the expression of CX3C chemokine receptor 1 (CX3CR1), the receptor for CX3CL1, in all spinal metastases samples showed negative staining.

**Materials and Methods:**

Cancellous bone in the spine from patients with and without spinal metastases was collected for mRNA microarray study, and then differentially expressed mRNAs related to chemokines were further confirmed by real-time polymerase chain reaction. Enzyme linked immunosorbent assay was used to detect the serum level of the selected chemokines and immunohistochemical staining was used to detect the expression level of corresponding receptors in tumor.

**Conclusions:**

Our present study showed that CX3CL1 is associated with the process of spinal metastases from different primary cancers.

## INTRODUCTION

It is estimated that more than 1.6 million new cases of malignant tumor were diagnosed in the United States in 2015 [1]. As a result of complications from metastases, about half of these patients eventually die from such diseases. Following the lung and liver, bone is the third most common site of metastases [2]. According to data from autopsy studies, as many as 50% of cancer patients are found to have spinal metastases [3], which also confirms that the spine is the most common site for skeletal metastases.

The ways through which cancer cells spread to the spine include blood dissemination, direct extension or invasion, and cerebrospinal fluid seeding. The vascular sinusoids lined with endothelial cells in the vertebra have fenestrae of 60 Å diameter and lack a basement membrane [4]. Accompanied with the low velocity of blood stream, cancer cells tend to bypass the endothelial layer and adhere to a cancellous substance. In addition, according to the concept of “Seed and Soil” theory [5], there is a close relationship between the selected metastatic cells and the vertebral microenvironment. During our clinical practice, it is noteworthy not only that highly aggressive cancers, such as liver and lung cancers, transfer to the spine, but also that less aggressive cancers induce spinal metastases, such as breast and prostate cancers. Thus, it is reasonable to speculate that some cytokines in the vertebral microenvironment are associated with this process.

Chemokines are a family of small, 8–10-kDa inducible cytokines. Because of the position variation of highly conserved cysteine residues in their primary structure, chemokines are subdivided into four subgroups: C, CC, CXC, and CX3C [6]. Given the effects of chemokines on chemotaxis of different cancer cells, we postulate that some of these molecules may mediate the processes of spinal metastases that are involved in different kinds of cancer.

The goal of the present study was aimed at identifying certain chemokines that stimulate a chemotactic response to the spine in different cancers. For this reason, we employed mRNA microarray and Real-Time polymerase chain reaction (RT PCR) to evaluate the expression of chemokines in para-carcinoma cancellous bone and normal cancellous bone. CX3C chemokine ligand 1 (CX3CL1) was chosen for further study. Therefore, we compared the serum concentration of CX3CL1 between metastatic spinal tumor groups and the normal control group. In addition, we detected the expression of CX3C chemokine receptor 1 (CX3CR1), the receptor for CX3CL1, in metastatic spinal tumor samples through the method of immunohistochemical staining.

## RESULTS

### Differentially expressed chemokine mRNAs and clustering analysis

**Figure 1:**
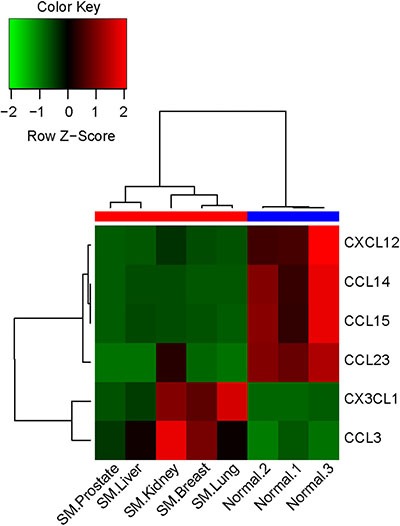
Heatmap of the selected differentially expressed chemokines between the two groups The horizontal axis represents the samples from the two groups (red: metastatic spinal tumors group; blue: normal control group). The chemokine names are shown on the right vertical axis. SM, spinal metastases.

The differentially expressed mRNAs related to chemokines between the two groups were identified using a human mRNA microarray. A total of 6 differentially expressed chemokines were identified, including four down-regulated and two up-regulated chemokines (Table 1). The expression levels of CX3CL1 and CCL3 were significantly increased in the metastatic spinal tumors group; this group had significantly lower expression levels of CCL14, CXCL12, CCL15, and CCL23 compared with patients in the normal control group. Furthermore, hierarchical clustering analysis indicated that there were clear differences between the two groups according to the expression levels of these six differentially expressed chemokines (Figure 1).

**Table 1:** The differentially expressed mRNAs of chemokines between the two groups

	Name	log2 fold change	*P* value
Up-regulated	CX3CL1	4.63	0.01469
	CCL3	1.56	0.00861
Down-regulated	CCL14	−3.74	0.00012
	CXCL12	−3.65	0.00398
	CCL15	−3.59	0.00028
	CCL23	−2.70	0.01383

### Validation of differentially expressed chemokines

**Figure 2:**
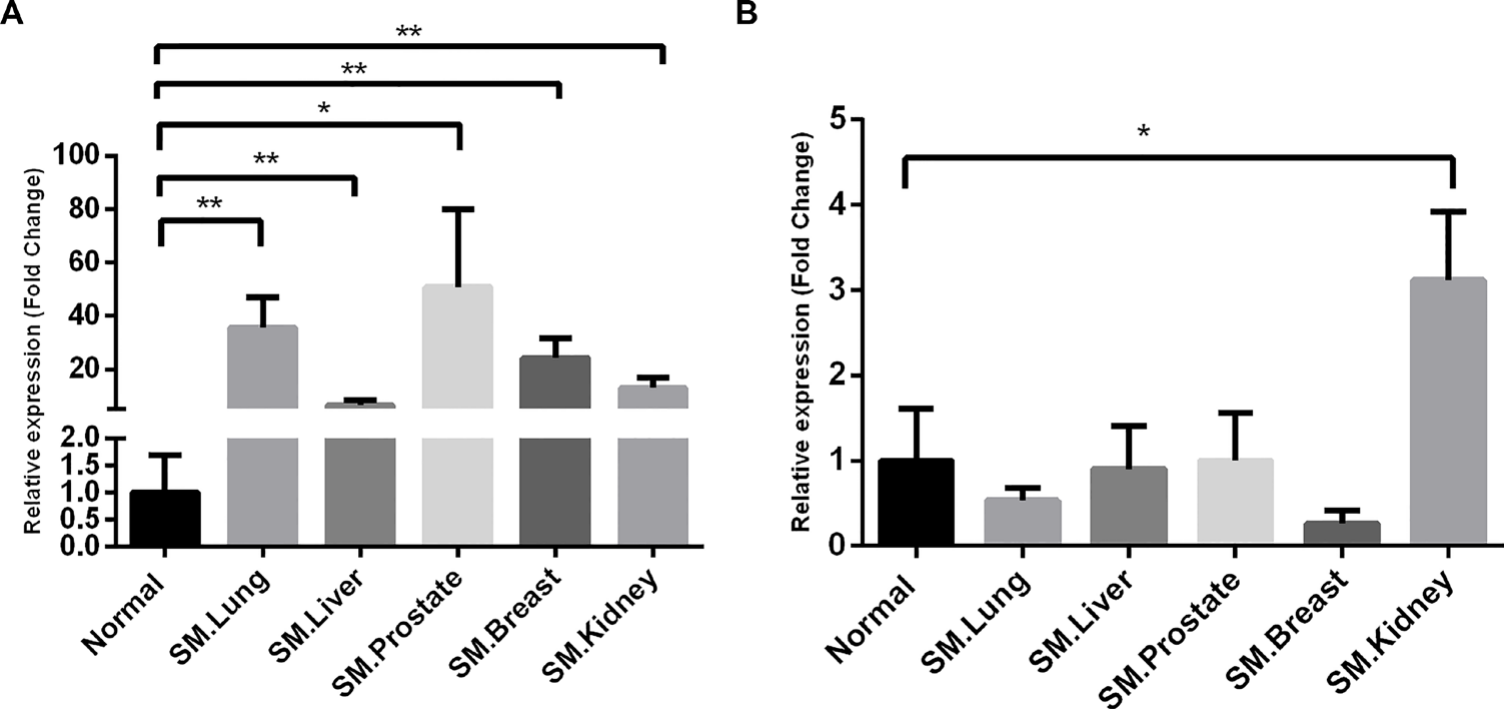
Validation of the selected chemokines by RT PCR β-actin was used for normalization. Data are expressed as the fold change over the normal control group, and represent the mean ± SD of three independent experiments. (**A**) CX3CL1; (**B**) CCL3. SM, spinal metastases. *P < 0.05; **P < 0.01.

To validate the reliability of the expression profile obtained by microarray analysis, we performed RT PCR for the two upregulated chemokines, CX3CL1 and CCL3, in an independent cohort of samples from the two groups. The RT PCR revealed that CX3CL1 was upregulated with significant differences in all of the metastatic spinal tumors groups (Figure 2A). However, the expression levels of CCL3 was only significantly increased in the group with kidney cancer (Figure 2B). These findings suggest that CX3CL1 may potentially contribute to the spine preferential metastases of cancer cells.

### Serum levels of CX3CL1

**Figure 3:**
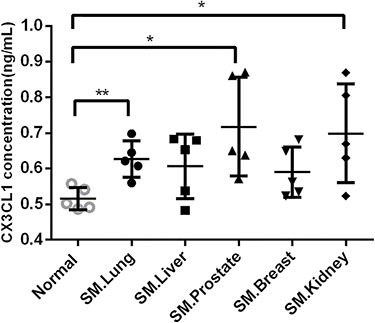
Serum levels of CX3CL1 in the different groups ELISA demonstrated that serum samples from patients with lung cancer (*P* < 0.01), kidney cancer (*P* < 0.05), and prostate cancer (*P* < 0.05) contained significantly higher levels of CX3CL1 compared with the serum samples from patients in the normal control groups. SM, spinal metastases. *P < 0.05; **P < 0.01.

Figure 3 shows the CX3CL1 concentrations in the different groups. The level of CX3CL1 in the serum samples from the metastatic spinal tumors was 0.63 ± 0.05 ng/ml in lung cancer, 0.61 ± 0.08 ng/ml in liver cancer, 0.58 ± 0.06 ng/ml in breast cancer, 0.69 ± 0.13 ng/ml in kidney cancer, and 0.71 ± 0.12 ng/ml in prostate cancer. Compared with CX3CL1 in the serum samples from the normal control groups (0.51 ± 0.03 ng/ml), serum samples from patients with lung cancer (*P* < 0.01), kidney cancer (*P* < 0.05) and prostate cancer (*P* < 0.05) contained significantly higher levels of CX3CL1, whereas those from patients with spinal metastases from liver cancer and breast cancer had a tendency to contain higher levels of CX3CL1 but without significance.

### CX3CR1 expression in metastatic spinal tumor tissues by immunohistochemical staining

**Figure 4:**
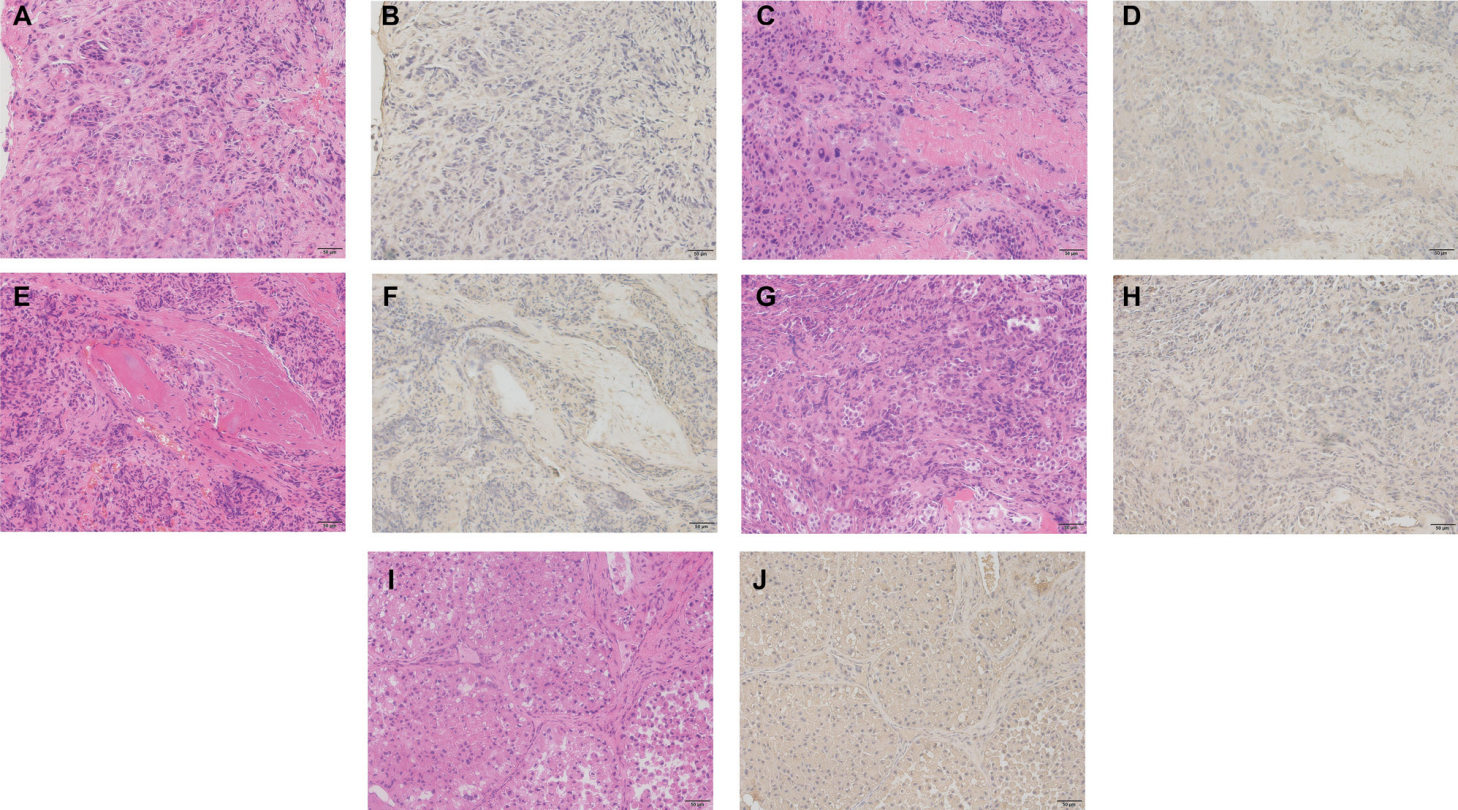
Immunohistochemical staining for CX3CR1 expression in metastatic spinal tumor tissue sections (**A**) and (**B**) show the hematoxylin eosin (HE) staining and immunohistochemical staining in metastatic spinal tumor from lung cancer; (**C**) and (**D**) show HE staining and immunohistochemical staining in samples from liver cancer; (**E**) and (**F**) showed HE staining and immunohistochemical staining in samples from prostate cancer; (**G**) and (**H**) showed HE staining and immunohistochemical staining in samples from breast cancer; (**I**) and (**J**) showed HE staining and immunohistochemical staining in samples from kidney cancer. Original magnification × 200 (scale bars 50 μm).

Immunohistochemical studies were carried out to detect CX3CR1 protein expression patterns in metastatic spinal tumor tissue sections. Results showed that CX3CR1 expression was negative in all of the metastatic spinal tumor tissues (Figure 4). The lack of staining in human tissue samples from the heart and the strong signal detected in samples of the human lung confirmed the specificity and validity of the CX3CR1 antibody used in this study (Supplementary Figure 1 online).

## DISCUSSION

In this study, we propose that CX3CL1 is associated with the process of spinal metastases derived from different primary cancers, which coincides with previous findings that CX3CL1 exhibits chemotaxis of different cancer cells [7–9].

Previous studies have implicated the expression of the plasma membrane–associated form of CX3CL1 in osteoblasts, mesenchymal, and stromal cells. In the present study, we found the elevated mRNA expression of CX3CL1 in para-carcinoma tissue. Furthermore, the serum level of CX3CL1 was also increased in patients with spinal metastases from the lung, kidney, and prostate. Based on these studies and our observation, it is plausible to hypothesize that the endothelial cells lining the bone marrow sinusoids express membrane-associated CX3CL1, which can provide viable docking sites for circulating CX3CR1-expressing cancer cells. On the other hand, the membrane-associated CX3CL1 can be shed into bone marrow and blood. Therefore, a possible concentration gradient of this chemokine is formed, which is able to chemo-attract CX3CR1-bearing cancer cells from the blood circulation into the spine. By analogy with its role in the leukocyte adhesion [10, 11], CX3CL1 obviates the need for both the association with proteoglycans and other adhesion molecules to bind CX3CR1-expressing cancer cells rapidly and with high affinity. In addition to the fact that CX3CL1 itself functions as an adhesion molecule, its cognate receptor, CX3CR1, can also enhance the avidity of integrin binding to its ligands through G proteins [12]. However, we did not detect the expression of CX3CR1 at the tissue level in the metastatic spinal tumor. This phenomenon may be ascribed to epithelial-mesenchymal transition (EMT) [13]. Because the cell characteristics are changed during EMT, it is possible that CX3CR1 is only expressed in mesenchymal cancer cells, which are prone to invasion and metastases. On the other hand, epithelial cancer cells are considered to be a more suitable form for cancer development over a long time, such as colonization in the spine. Because our samples from the spine have grown for a relatively long time, it is not so surprising that these cancer cells fall within the classification of epithelial cancer cells, and were lacking in CX3CR1. In addition, another possible explanation is that cancer cells with lack or low levels of expression of CX3CR1 are more like to spread to metastatic sites rather than stay in primary site [14]. Therefore, higher concentration of CX3CL1 in bone marrow and serum are required to recruit cancer cells with low CX3CR1 expression. Alternatively, other chemotactic stimuli rather than elevated CX3CL1 in bone marrow and serum recruit such cancer cells.

Of note, CX3CL1 has been shown to contribute to the growth of tumors [15]. It was recently reported by Tsang that CX3CL1 could have a pro-tumor role in breast cancer, because breast cancers with a high expression of CX3CL1 were found to have poorer overall survival [16]. However, there is much debate concerning the relevance of CX3CL1 to cancer growth and progression. Some studies concluded that the greater the CX3CL1 expression, the better the prognosis, which is in accordance with the previously suggested role of CX3CL1 in enhancing anti-tumor immunity. This is supported by experiments in orthotopic implantation of lung cancer, where the antitumor effects of CX3CL1 were derived from natural killer cell activities [17]. As proposed by Park and colleagues [18], patients with high CX3CL1 expression had a significantly better disease-free and overall survival than those with low CX3CL1 expression as a result of the enhanced recruitment of immune cells. In addition, the CX3CL1 expression was found to be correlated with good prognosis in colorectal carcinoma [19]. Here we identified CX3CL1 as the common chemokine-mediating spine metastases, which is independent of tumor type. As observed in the previously mentioned studies, CX3CL1 has two opposing effects on tumor progression. The occurrence of one of these two effects may depend on the amount of CX3CL1 present or the ability of tumors to divert CX3CL1 functions to favor their development through several mechanisms. These mechanisms include direct growth activity, stimulation of angiogenesis, control of spreading and metastases, and interference with the recruitment of different leukocyte populations [20].

It is true that tumor metastases are extremely complex, and CX3CL1 needs other molecules to work in a coordinated fashion to induce spinal metastases. However, this study may lead to translational clinical utility. For example, we can explore the potential effect of novel treatment strategies based on CX3CL1 expression modulation. Indeed, many experimental models have been established to explore the potential regimen targeting to other members of the chemokine family. Several lines of evidence in this field have been shown to be capable of inducing tumor regression and inhibiting metastases [21–23].

The main limitation of our study was the small sample size, because suitable cases are rare. Although up to 50% of spinal metastases require some form of treatment, only 5% to 10% require surgical management [24]. Even fewer patients eventually had surgery because of the high cost of operation. However, in view of the severe complications and impaired life quality as a result of the spinal metastases, there is still an important need for a regimen for this disease. Little effort has so far been directed toward the spinal metastases focusing on the spine itself, especially the bone microenvironment. It is therefore valuable to conduct research from this new perspective.

Collectively, the accumulating evidence from the literature and our data suggest that CX3CL1 is a critical component in the development of spinal metastases in diverse tumors, including the lung, prostate, kidney, and so on. However, the latent molecular mechanism remains to be elucidated, and there is a lack of conclusive evidence for a determinant role of the CX3CL1 in spinal metastases. These experiments are currently being conducted in our laboratory, and we are convinced that it is valuable to further delineate the pathophysiological role of CX3CL1 and its clinical therapeutic potential.

## MATERIALS AND METHODS

### Patients and specimen collection

Two groups of patients were enrolled: a metastatic spinal tumors group and a normal control group. Specifically, the metastatic spinal tumors group contained lung cancer, breast cancer, liver cancer, prostate cancer, and kidney cancer, each of which included 6 cases. These patients received total en bloc spondylectomy at Zhongshan Hospital from January 2012 to December 2015. All patients had a histologic diagnosis. The normal control group consisted of eight patients who received spine surgery at Zhongshan Hospital because of non-tumor diseases. The tumor and para-carcinoma cancellous bone from the metastatic spinal tumors group were collected, whereas the normal cancellous bone from the normal control group was collected. At the same time, preoperative serum from the both groups was obtained. All the above procedures were deployed with the signed consent form the patients and approval of the Ethics Committee of the Zhongshan Hospital, and all the experiments were carried out in accordance with relevant guidelines and regulations.

### RNA isolation

For RNA extraction of cancellous bone, we used RNeasy Protect Minikit (Qiagen, Carlsbad, CA, USA) according to the manufacturer's instructions. The RNA concentration was detected by a NanoDrop 1000 Spectrophotometer (NanoDrop, Wilmington, DE, USA), and its integrity was assessed through a Bioanalyzer 2100 (Agilent, Santa Clara, CA, USA). The samples with A260/A280 ratios > 1.9 and RIN values > 8.0 were the premises for further use.

### Microarray analysis and data processing

One sample of cancellous bone from metastatic spinal tumor with each different primary cancers and three samples of cancellous bone from normal controls were used for mRNA microarray analysis. The gene expression profiles of these samples were determined using Illumina HumanHT-12_V4 BeadChip arrays (Illumina, San Diego, CA, USA), which contains more than 47,000 probes, including gene probes or probesets derived from the NCBI, RefSeq, and UniGene databases. According to the manufacturer's protocol, the samples finally hybridized to the bead arrays at 55°C for 18 hours and were then scanned using an Illumina iScan reader (Illumina, San Diego, CA, USA). The initially scanned array intensity data were analyzed using the Illumina Genome Studio Gene Expression Module (v1.1.1) (Illumina, San Diego, CA, USA). Genes related to chemokines were selected and further analyzed. A *t*-test analysis was used to identify the differentially expressed genes between the two groups. The raw *P* values were adjusted into false discovery rate (FDR) using the Benjamin and Hochberg method [25]. A cluster analysis was used to group the cases into clusters based on the selected genes with FDR < 0.05 and | log_2_ fold change |>1.

### RT PCR

For data verification, samples except for microarray analysis were included as an independent cohort. Therefore, there were five sample of cancellous bone from metastatic spinal tumor with each different primary cancers and five samples of cancellous bone from the normal control group. The relative quantification of selected differentially expressed mRNAs of chemokines was performed by RT PCR reaction with the SYBR Green PCR Kit (Takara, Japan) using the ABI 7500 Real-Time PCR System (Applied Biosystems, Foster City, CA, USA). All the primers were purchased from Sangon Biotech Co. Ltd. (Shanghai, China) (Supplementary Table 1 online). The annealing temperature was 60°C, and the amplification cycles were 40. The PCR results were quantified using the 2^−ΔΔct^ method against β-actin for normalization. Each sample was tested in triplicate for analysis of relative gene expression, and melting curve analyses were conducted to confirm generation of the specific PCR product.

### ELISA assay for CX3CL1

Arterial blood was sampled from individuals included in the microarray data verification by RT PCR at the time of the pre-operation. Therefore, there were 5 serum samples from the metastatic spinal tumor group with each of the different primary cancers and five serum samples from normal control group. Blood samples rested 60 minutes to clot at room temperature before centrifugation for 15 minutes at 1000G. After centrifugation, serum samples stored at −80°C until analysis were conducted. The soluble form of CX3CL1 was detected in serum using the Quantikine Elisa Kit (R&D Systems, Minneapolis, MN, USA) according to the manufacturer's protocol. Each sample was tested in triplicates.

### Immunohistochemistry

In brief, immunodetection of CX3CR1 in metastatic spinal tumor tissue sections was performed using rabbit antihuman CX3CR1 (Abcam, Cambridge, MA) as the primary antibodies. Sections were incubated with the primary antibody (1:100) overnight at 4°C after heat-mediated antigen retrieval, and then incubated with peroxidase-labeled anti-rabbit antibody at 37°C for 60 minutes. Slides were then processed using 3,3-diaminobenzidine chromogen solution and counterstained with hematoxylin. To confirm the specificity and validity of the CX3CR1 antibody, we employed human tissue sections from the heart as negative control because of its lack of staining, and we used tissue sections of human lung as positive control because of the existence of staining according to previous studies [9].

### Statistical analysis

Experimental results were expressed as mean ± SD and data were analyzed using SPSS 20 statistics software (SPSS Inc., Chicago, IL, USA). Statistical significance between the two groups was determined with the *t*-test, and a *P* value < 0.05 was assumed as statistically significant.

## SUPPLEMENTARY MATERIALS FIGURES AND TABLES


