# Supplementary material for: CX3CL1: a potential chemokine widely involved in the process spinal metastases
Source: Oncotarget. 2017 Jan 20;8(9):15213–9. doi: 10.18632/oncotarget.14773 (PMC5362480; doi:10.18632/oncotarget.14773)
Supplement: Supplementary file 1 [file oncotarget-08-15213-s001.pdf]

CX3CL1: a potential chemokine widely involved in the process spinal metastases

Supplementary Materials

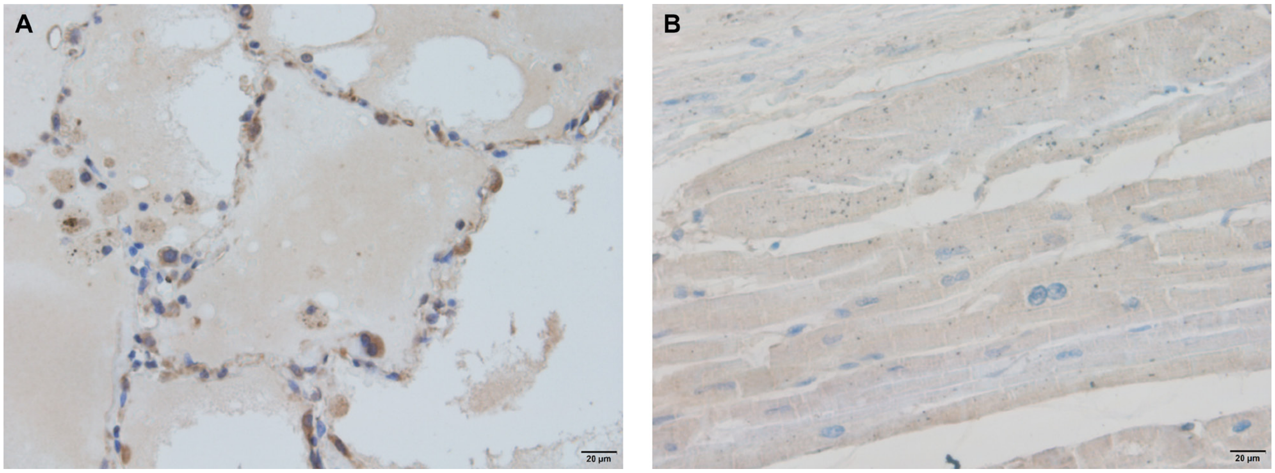

Supplementary Figure 1: Immunohistochemical staining for CX3CR1 expression in the lung and heart. The specificity and validity of the primary antibody against CX3CR1 was validated using tissue section from the lung (**A**), which expresses CX3CR1, and the heart (**B**), which does not express CX3CR1. Original magnification × 400 (scale bars 20 μm).

Supplementary Table 1: Primers used for RT PCR

Gene	Primer sequences (Forward/Reverse)
CX3CL1	5′-CGGCAAACGCGCAATCATC-3′<br>5′-TTCTCGAAGGTGCCGCCATTT-3′
CCL3	5′-CAACCAGTTCTCTGCATCACTTG-3′<br>5′-GCTCGTCTCAAAGTAGTCAGCTA-3′
β-actin	5′-CAACCGCGAGAAGATGACCC-3′<br>5′-GAGGCGTACAGGGATAGCAC-3′
